# Correlation between BAC and abdominal muscle alcohol concentration: a promising parachute matrix in cases of severe injury

**DOI:** 10.1007/s12024-026-01212-x

**Published:** 2026-03-05

**Authors:** Pascale Basilicata, Angela Simonelli, Mariagrazia Marisei, Maurizio Municinò, Rossella Guadagni, Angela Silvestre, Maria Pieri

**Affiliations:** 1 Department of Advanced Biomedical Sciences, University of Naples “Federico II”, Via S. Pansini 5, Naples, 80131 Italy; 2 Legal Medicine Division, Local Health Authority (ASL) Napoli2Nord, Naples, Italy

**Keywords:** Toxicological analyses, Post-mortem alcohol concentration, Muscle, Alternative specimens, BAC estimation, Severe injury

## Abstract

Ethanol represents the most abused drug worldwide, and among the most frequently evidenced in toxicological analysis on drug-induced/related deaths. When a blood sample is not available in a death characterized by severe trauma an alternative matrix is needed for an estimation of impairment. Presented is the correlation of postmortem blood and rectus abdominis muscle ethanol concentration. Ethanol concentrations in both blood (BAC) and muscle were determined by head space gas chromatography/mass spectrometry. Twenty-nine forensic cases with positive BAC were selected for the correlation study. Circumstances and time since death were known in all selected cases. Muscle analyses evidenced a linearity of the analytical response within the range (0.03–0.9) mg_ethanol_/g_muscle_). Quantification of three QC samples (0.15–0.3 and 0.6 mg_ethanol_/g_muscle_) through aqueous calibrators was superimposable to matrix-based one. The method was validated, by assessing specificity, recovery from biological matrix (92.5%), sensitivity (LOD of 0.15 mg_ethanol_/g_muscle_, LLOQ 0.03 mg_ethanol_/g_muscle_), bias of quantitative analyses was between 3.5% and 6.1%. ANOVA F-test was used to determine within-run and between-run precision, both resulted minor than 20%. A linear correlation between BAC and ethanol muscle levels was obtained for selected post-mortem samples and correlation equation (y = 0,5837x + 0,0353; R² = 0,8661) was used for BAC estimation in three forensic cases for which blood was not available. Rectus abdominis muscle was demonstrated as a good candidate to estimate blood alcohol concentration and impairment at the time of fatality.

## Key points

Ethanol represents the most abused substance worldwide.

Estimation of impairment when a blood sample is not available is challenging.

Presented is the correlation of post-mortem blood and rectus abdominis muscle ethanol concentration.

Rectus abdominis muscle is good candidate to estimate blood alcohol concentration and impairment at the time of fatality.

## Introduction

Ethanol intake alone or in combination with other drugs of abuse represent a risk factor for severe injury, mostly roadside [1, 2]. Ethanol impairment is among the top ten causes of death in all countries except for high-income ones [3–5]. Compared to the previous year, in 2020 there was an approximately 14% increase in people killed in motor vehicle crashes involving alcohol-impaired drivers, accounting for 30% of all traffic-related deaths in the United States [6]. Alcohol is also responsible for many other indirect alcohol-related harms, due to behaviours associated with acute intoxication states, such as risky sexual behaviour, work-related injuries, violent incidents, drunk driving [7]. In the case of drunk driving, the risk of a road traffic crash starts at low levels of blood alcohol concentration (BAC) and increases significantly when the driver’s BAC exceeds 0.4 g/L [3], with a remarkable positive correlation between BAC and injury severity in road accident victims [8].

Peripheral blood, bladder urine, and vitreous humor are the preferred biological fluids for ethanol postmortem detection, and femoral blood is the matrix of choice to derive a value that is not -or minimally- influenced by redistribution and/or postmortem formation [9]. A literature review was devoted to developing of guidelines for the sampling and analysis of biological samples for toxicological purposes [10]. In decomposed bodies the need to discriminate between ante-mortem intake and post-mortem ethanol formation makes it necessary to find “unconventional specimens” [11], such as liver, brain, spleen, bone marrow, cerebrospinal fluid, bile, synovial fluid and skeletal muscle. The same need may arise in deaths characterized by high traumatic injury, where blood is not always available. Toxicological and medico-legal evaluations in these cases can be extremely complex due to the lack of an adequate biological sample (i.e., cases of severe traumatisms with only human remains available, severe hemorrhagic shock, etc.).

In cases characterized by a high *vis lesiva* and in which the decomposition phenomenon has not occurred, the problem is not so much to identify the most suitable matrix for limiting the bias of postmortal ethanol production, but to identify an alternative matrix that yields a correlation with the degree of impairment “at death” correlating data obtained on alternative matrix to BAC. A literature review revealed few studies aimed at verifying the possibility of using muscle as an alternative to blood in ethanol post-mortem detection [11–16]. Although limitations due to sample variabilities (especially related to the cadavers conditions at autopsy), available studies evidenced the possibility of skeletal muscles (psoas, thigh, femoral quadricep) as an alternative matrix. Madro and Buszewicz also evidenced the appropriateness of analysing femoral muscle samples as a valid alternative to blood, obtaining a good correlation between ethanol levels in peripheral blood [12]. Iffland and Staak compared results from psoas and femoral quadriceps with BAC. A strong correlation between alcohol levels in femoral vein blood and thigh muscle tissue was obtained, thus showing the appropriateness of using thigh muscles when femoral blood is not available [13]. Within this context, the aim of the present study is to assess the possibility of using rectus abdominis muscle as an alternative matrix to blood for estimating BAC in deaths characterized by a high degree of traumatism.

## Materials and methods

Ethanol and isopropyl alcohol (used as internal standard, I.S.) were from Merck (Darmstadt, Germany).

Head-space gas chromatographic/mass spectrometric (HS-GC/MS) analyses, using *full scan* acquisition mode, were performed on an HP6890 series gas chromatographer attached to a HP7694E autosampler and a 5973 single quadrupole mass spectrometer. Chromatographic separation was accomplished by a PoraPlot Q (2.5 m particle trap, 27.5 m x 0.25 mm x 0.8 μm) column from Agilent (Santa Clara, CA, US). Data were analyzed using the MSD Chemstation, software (D.02.0.275 version), from Agilent.

### Study design

Twenty-nine rectus abdominis muscle samples from as many subjects were collected at autopsy and stored at -20 °C until analysis. Formal approval was obtained from the Public Prosecutor’s Office. The subjects’ inclusion criteria were availability of both muscle and peripheral blood, absence of metabolic and/or pathological conditions influencing physiological ethanol absorption/metabolism, absence of putrefactive phenomena at autopsy and a positive BAC. In particular, all samples resulted negative to acetone, thus allowing the use of isopropyl alcohol as I.S [17]. Six muscle samples from subjects with negative BAC were used as matrix blank. Ethanol concentration values obtained from twenty-nine selected muscle samples were plotted against BAC and correlation equation was used for BAC estimation in three cases (A, B and C) for which blood was not available. Mean values obtained from at least three sample replicates were considered for both muscle ethanol and BAC in all analyzed samples. Details of circumstances of death for cases A, B and C are briefly reported below.

- *Case A*. A 28-year-old woman passed away after falling off the motorcycle she was riding as a passenger. Suddenly after the fall the woman was hit by at least three cars. The external examination revealed several traumatic injuries, including a cracked skull, a detached right lower limb from the trunk, and thoracic and abdominal organs. Abdominal muscle residues and lung tissue were the only available samples.
- *Case B*. A 19-year-old man with suicidal aims died after being hit by a train. On external examination, the corpse was missing the lower limbs (found on the sidewalk) and the torso (found on the roadbed next to the track). No blood or other fluid samples were available.
- *Case C*. A 45-year-old man died after multiple car investment. Several cars hit the man while he was walking near a highway junction, resulting in severe injury. No blood or other fluid samples were available.

### Blood ethanol concentration

BAC determination was performed on peripheral blood samples according to a previously published procedure [18]. Briefly, 300 µL blood aliquot, fortified with 100 µL of 1 mg/mL aqueous I.S. solution, were putted in a 20 mL head space vial and immediately crimped. The vials were kept at 150 °C for fifteen minutes and then 100 µL of the head-space was injected. Isothermal separation was achieved at 150 °C held for 9.5 min. Retention times were 4.6 min for EtOH and 5.8 min for I.S.

Quantitative analyses were based on six non-zero standard calibrators in the range (0.1-3.0) g/L. For sample preparation, 300 µL of each aqueous EtOH solution, fortified with 100 µL of 1 mg/mL aqueous I.S. solution, were put in a 20 mL HS vial. Three quality control (QC) samples were independently prepared at 2.0, 1.0 and 0.5 g/L.

### Muscle sample preparation

Aliquot of 1 g of finely cut abdominal muscle sample was put into a 20 mL HS-GC vial, spiked with 100 µL of 1 mg/mL aqueous I.S. solution, and immediately crimped. Ethyl alcohol determination was performed as described for blood. Matrix-based calibrators in the range (0.03–0.9) mg_ethanol_/g_muscle_ and three QC samples (0.15–0.3 and 0.6 mg_ethanol_/g_muscle_) were prepared by adding proper amount of the ethanol standard solution to 1 g aliquot of finely cut ethanol-free muscle sample; each sample was fortified with 100 µL of 1 mg/mL aqueous I.S. solution for the subsequent analysis.

### Validation of the analytical procedure

Analytical procedure was validated according to ANSI/ABS Guidelines [18]. Quantitative analytical response obtained through matrix-based calibrators and aqueous standard ones were compared, to verify their equivalence in terms of accuracy. All samples were analyzed in triplicate. To such an extent, three 1 g aliquots of muscle were finely cut and fortified with 100 µL of 1 mg/mL aqueous I.S. solution. The analytical procedure for muscle ethanol determination was validated, by determining specificity, linearity, limit of detection (LOD) and lower limit of quantitation (LLOQ), recovery, intra- and inter-day precision, accuracy.

#### Specificity 

Six muscle samples from subjects with negative BAC were analyzed in HS-GC/MS to determine possible endogenous interferents. Samples resulted negative were used as blank muscle samples for subsequent analyses.

#### Linearity and reproducibility

 Procedure responses were determined for six standard aqueous calibrators and matrix-based ones on six non-consecutive days. Linear fitting was applied, and correlation equations were compared in terms of the intercept on the *y*-axis and the angular coefficient.

#### LOD and LLOQ

 Mean ethanol concentration obtained for six matrix-blank samples with associated standard deviation (SD) were used to determine limit of detection and of quantification, as 3*SD and 5*SD for LOD and LLOQ, respectively.

#### Recovery

Ethanol recovery from muscle sample was obtained as ratio between angular coefficients of the matrix-base calibration curve with standard aqueous one.

####  Bias and precision

Bias was calculated by using QC samples, each prepared and analyzed in triplicate on five different days. Precision in terms of coefficient of variation was determined in both within-run and between-run analyses using the three quality control samples, each prepared and analyzed in triplicate. Within-run and between-run precision were evaluated for QC samples prepared in triplicate on five different days. One-Way Analysis of Variance (ANOVA) single test was used to calculate combined within-run precision and between-run precision for the 0.05 significance level [19].

## Results

### Muscle ethanol quantification

**Fig. 1:**
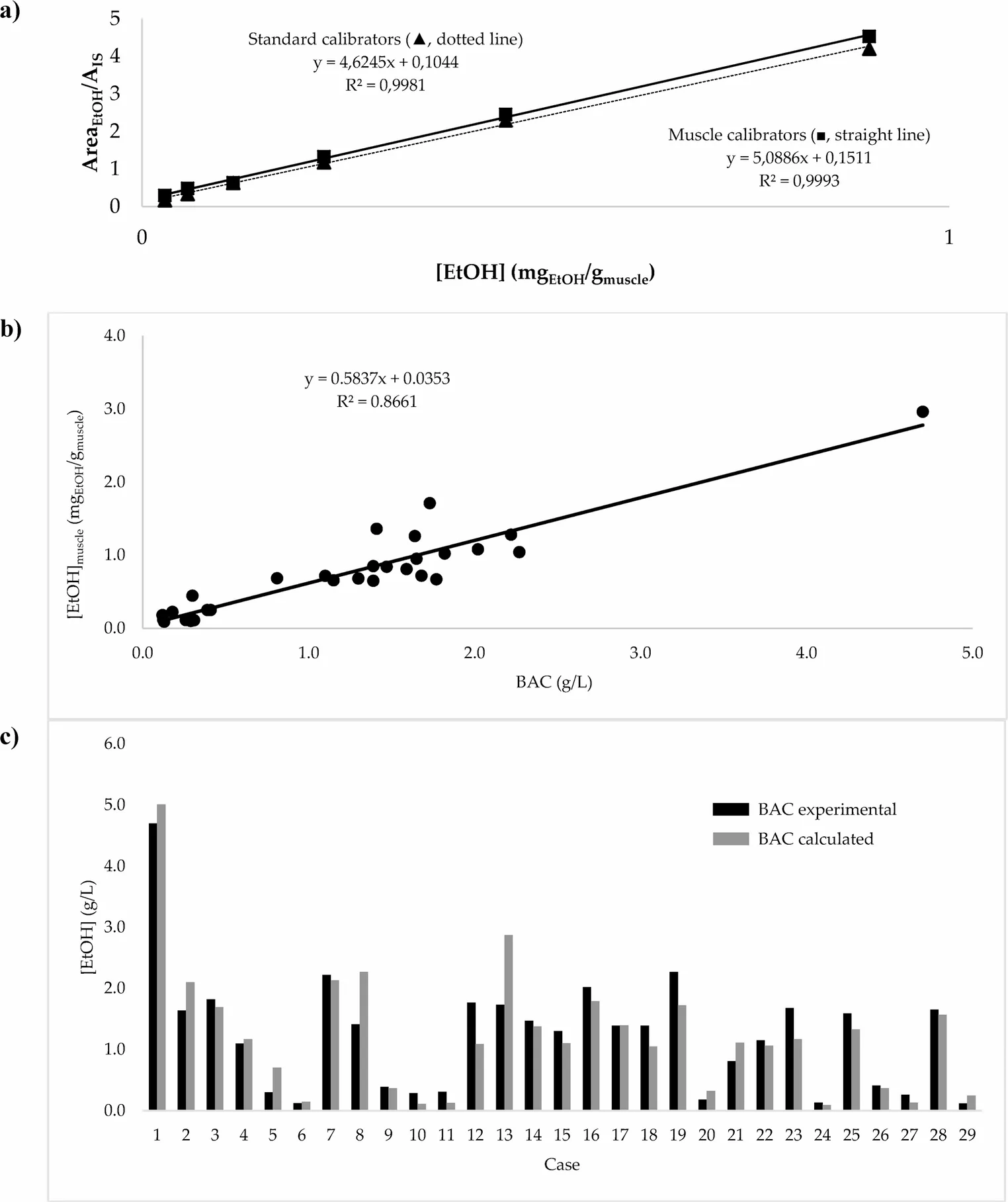
Panel **a**) Comparison between muscle based-calibration curve (■, straight line) and standard aqueous one (▲, dotted line). Panel **b**) Correlation between muscle ethanol concentration and BAC. Panel **c**) Comparison between BAC experimental value - black bar - and calculated ones considering all study samples - grey bars

Matrix-based and aqueous calibrators were compared to define the quantification procedure for muscle ethanol determination. Figure 1 (panel a) shows an example of calibration curves obtained using matrix-based (■, straight line) and aqueous calibrators (▲, dotted line). The two curves showed similar angular coefficients and y-axis intercept, evidencing the substantial equivalence between matrix-based and aqueous calibrators. The correlation equations of the two calibration curves were used to back-calculate muscle ethanol concentration, obtaining almost superimposable results for QC quantification. Consequently, standard aqueous calibration curve was used for validation study and further determination of muscle ethanol concentration in all enrolled samples.

### Validation parameters

No interfering peaks at the ethanol retention time were present in any of the six blank muscle samples. The analytical response was evaluated on six non-consecutive days. Data shown a linear correlation within considered calibration range (0.03–0.9) mg_ethanol_/g_muscle_. Calibration curves presented correlation coefficients R2 > 0.99. The analytical procedure was characterized by a LOD of 0.15 mg_ethanol_/g_muscle_; the LLOQ was 0.03 mg_ethanol_/g_muscle_. Ethanol recovery from muscle samples was higher than 92.5%. Bias, within-run and between-run precision (expressed as CV%) calculated at three QC sample levels (QC1, QC2 and QC3 at concentration of 0.15, 0.3 and 0.6 mg_ethanol_/g_muscle_, respectively) are reported in Table 1. Bias varied within 3.5% (QC3) to 6.1 (QC1); within-run precision was below 20% for all QC levels; between-run precision varied from 2.5% (QC3) and 7% (QC1). One-Way Analysis of Variance (ANOVA) single test was used to verify the null hypothesis (absence of statistically significant differences between the mean values measured within and between runs) and to calculate combined within-run precision and between-run precision [12]. F-test for the 0.05 significance level was performed for the three QC levels, each quantified in triplicate on five different days. Results of F-test (FQC1 = 1.06, FQC2 = 1.87, FQC3 = 0.90) were less than the F critical value for the 0.05 significance level (Fcrit = 3.478). The combined between-run CV% were 3.27%, 1.22%, and 1.27% for QC1, QC2, and QC3, respectively.

### Correlation between BAC and muscle ethanol concentration

Twenty-nine abdominal muscle samples collected from deceased with non-negative BAC were analyzed for tissue ethanol. None of selected samples resulted positive to acetone, thus allowing the use of isopropyl alcohol as internal standard for ethanol quantification. Obtained values (mean of at least three independent replicates) are reported in Table 2. Muscle ethanol concentration (*y*) was plotted against BAC (*x*) and a linear fit was applied to experimental values, obtaining a correlation equation of y = 0.5837x + 0.0353, R² = 0.8661 (Eq. 1, Fig. 1, panel b). Figure 1, panel c, reports BAC experimental values (black bars) and values calculated with Eq. 1 (gray bars).

**Table 1:** Calculated mean ethanol concentration, bias, within-run and between-run and precision obtained for muscle QC samples quantified through standard aqueous calibration curves. Each QC was analysed in triplicate on five non-consecutive days

	Calculated mean (mg_ethanol_/g_muscle_)	Bias (%)	Within-run Precision (%)	Between-run Precision (%)
QC1 (0.15 mg_ethanol_/g_muscle_)	0.16	6.1%	1.4%-13.2%	7.0%
QC2 (0.3 mg_ethanol_/g_muscle_)	0.32	5.1%	0.9%-3.9%	3.3%
QC3 (0.6 mg_ethanol_/g_muscle_)	0.62	3.5%	0.7%-4.2%	2.5%

Equation 1 was used to estimate BAC of cases A, B and C for which blood was not available. In case A analysis was repeated on a diluted sample to obtain a concentration within calibration curve range. Results reported in Table 3 evidenced as for case A ethanol blood level were greater than 3.0 g/L (3.22 g/L). Although datum represents an estimation, the particularly high BAC is in line with the defense hypothesis of a sudden accidental fall of the girl in consequence of her impaired state. Positivity found in case B has similarly positive consequences for train-driver. According to the defensive thesis, the accident was inevitable due to the man’s sudden appearance on train tracks. Finally, in case C positivity to alcohol found in abdominal muscle could support the thesis of recent alcohol intake by the man.

**Table 2:** BAC and muscle ethanol concentrations determined in enrolled study samples; value as mean of three independent replicates are reported with associated standard deviation (SD). Ratio between BAC and ethanol muscle concentration is reported in the last column

Sample	BAC (g/L)	SD	[EtOH]_muscle_(mg_EtOH_/g_muscle_)	SD	BAC/[EtOH]_muscle_
1	4.70	0.10	2.96	0.10	1.59
2	1.64	0.01	1.26	0.09	1.30
3	1.82	0.06	1.02	0.05	1.78
4	1.10	0.04	0.72	0.08	1.54
5	0.30	0.13	0.45	0.04	0.67
6	0.12	0.09	0.12	0.03	1.03
7	2.22	0.05	1.28	0.03	1.73
8	1.41	0.11	1.36	0.05	1.04
9	0.39	0.04	0.25	0.02	1.56
10	0.29	0.02	0.10	0.01	2.90
11	0.31	0.05	0.11	0.01	2.82
12	1.77	0.07	0.67	0.14	2.64
13	1.73	0.04	1.71	0.07	1.01
14	1.47	0.21	0.84	0.11	1.75
15	1.3	0.04	0.68	0.07	1.91
16	2.02	0.05	1.08	0.01	1.87
17	1.39	0.04	0.85	0.03	1.64
18	1.39	0.09	0.65	0.03	2.14
19	2.27	0.01	1.04	0.10	2.18
20	0.18	0.03	0.22	0.01	0.81
21	0.81	0.05	0.68	0.04	1.18
22	1.15	0.02	0.66	0.04	1.75
23	1.68	0.09	0.72	0.03	2.34
24	0.13	0.06	0.09	0.01	1.44
25	1.59	0.05	0.81	0.07	1.96
26	0.41	0.07	0.25	0.03	1.64
27	0.26	0.03	0.11	0.01	2.32
28	1.65	0.07	0.95	0.05	1.74
29	0.12	0.01	0.18	0.01	0.67

## Discussion

Ethanol is the most used/abused psychoactive substance and is frequently found in forensic evaluations. Subjects involved in car accidents and/or circumstances characterized by severe traumatism commonly result positive toward alcohol alone or in combination with other drugs of abuse. A positive correlation between injury risk and blood alcohol levels and other drugs intake has been reported [20–22]. Blood is the matrix of choice in post-mortem analyses aimed to verify a possible impaired state of the deceased that could have directly or indirectly caused death [23]. It follows that difficulties in evidencing an impaired state may arise when blood or other matrices of current/recent use (and for which a correlation between biological concentrations and effects is known [24]) are not available, with the consequent need for alternative matrices [25]. The possibility of performing “effective” toxicological analyses on an alternative matrix is related to the availability of a correlation between obtained results and the subject’ impaired state. Such a latter aspect is of primary importance in the forensic context due to its possible relevance in terms of liabilities of both the defendant and the victim. It must be emphasized that «performing a correct forensic evaluation is mandatory», the term “correct” means not only to derive an analytically accurate result but being able to correlate the toxicological result with the impairment degree.

**Table 3:** BAC estimation for cases A, B and C through correlation equation between muscle ethanol concentration and BAC reported in Fig. 1 (*y* = *0,5837x* + *0,0353*).

Sample	[EtOH]_muscle_(mg_EtOH_/g_muscle_)	BAC_estimated _(g/L)
A	1.92^a^	> 3.0 (3.22)
B	0.44	0.69
C	0.07^b^	<0.05

^a^quantitative value was obtained on 0.3 g of muscle sample spiked with 0.7 g of blank muscle matrix. ^b^quantitative value was obtained on 2.0 g of muscle sample

If assessing the correlation between blood levels and impairment is still challenging for several drugs of abuse [26–28], ethanol may be considered an exception due to the amount of correlation studies available in literature [29–33]. Despite this, assessing the subject’ ante-mortem ethanol impairment is still challenging when blood is not available. Within this context, the here presented study was aimed to verify the possibility of performing toxicological analyses on abdominal muscle, the only biological specimen available at autopsy in three cases of severe injury related to motorcycle accident (case A), suicide for stepping against a train (case B) and consequent to multiple car investment (case C). In all cases highlighting an eventual impaired state of the victims was crucial in defining the liability of the defendants (motorcycle, train and cars drivers for case A, B and C, respectively). Twenty-nine subjects with non-negative BAC and for which abdominal muscle was sampled at autopsy were selected and analysed to define a)the suitability of the matrix for toxicological analyses; ii)correlations between tissue ethanol concentrations and BAC.

The first part of the study allowed the definition and validation of the analytical procedure for muscle ethanol quantitative analysis through head space-gas chromatography coupled with mass spectrometry. The analytical response of matrix-based calibrators and quality control samples was similar to the one obtained for aqueous solutions. A linear response was obtained within the considered range (0.03–0.9) mg_ethanol_ /g_muscle_. During sample preparation, the muscle aliquots were not homogenized, to prevent samples’ heating and consequently to avoid eventual ethanol lost. The minimum sample preparation, together with the possibility of avoiding the use of matrix-based calibrators make the procedure extremely simple and less time- and money-consuming. Moreover, it must be highlighted as the use of standard calibrators let toxicologists to avoid forensic samples’ misuses, as *“the coroner*,* or a person or court with a similar responsibility in a jurisdiction*,* has the responsibility to determine the type of investigation conducted on the death and what tests are appropriate. Increasingly*,* this does not include research unless some prior specific permission has been sought and obtained”* [23]. This aspect is central in countries like Italy, where the coroner institute does not exist at all. In Italy the Prosecutor Office authorizes only analyses useful to elucidate the causes of death, and toxicological analyses cannot in principle involve the use of samples from other forensic cases if not for an ad hoc authorization.

The second part of the study was aimed to verify the existence of a correlation between BAC and muscle ethanol content. To such extent, thirty-six post-mortem cases with positive BAC and for which abdominal muscle was available were selected and analysed. Deceased with metabolic and/or pathological conditions associated with altered ethanol metabolism were excluded from the study. Bodies presenting a stomach rupture or in evident putrefaction state were not considered as well. Results of the study evidenced a good linear correlation between muscle ethanol concentration and BAC within the considered range.

The application of the correlation equation (y = 0.5837x + 0.0353) to derive BAC for cases A-C showed positive blood ethanol values with useful consequences on penal level, since the defence of the drivers argued that they were completely not guilty, the accidents being inevitable due to victims’ impaired state. This was particularly true in case A, where ethanol concentration higher than 3.0 g/L was estimated. The extent of alcohol induced alteration could reasonably explain the woman’s sudden fall from the motorcycle, excluding the motorcycle driver from criminal liability. Similar considerations can be derived for case B - the altered state of consciousness presented by the man makes the hypothesis of a sudden gesture more than probable, with no possibility for the train-driver to avoid the impact. Finally, positivity obtained for case C supported both the suicide hypothesis and the possibility that the man could not has probably realized the risks of walking near a highway junction. The positive consequences for the defendants are intuitive.

In general, ethanol tissue levels obtained in the present study are lower than BAC. Muscle ethanol concentrations lower than BAC were also reported by Christopoulos et al. in six subjects [34].

Garriott published results obtained for aorta blood and thigh muscle sample, obtaining comparable ethanol levels for BAC greater than 0.1 g/dL; muscle levels were higher than blood concentration when BAC was lower than 0.1 g/dL [16]. The Author attributed such differences to the condition of the sample studied: the group presenting similar concentration were considered to the *at or near the maximum ethanol concentration or may have been drinking alcohol at time of death* [16]. Individuals for which muscle ethanol levels were greater than blood were considered to be in a postabsorptive phase, with declining BAC and lagging muscle concentration (time needed for muscle ethanol to equilibrate with blood concentration differs between pre-absorptive and post-absorptive phases) [16]. The choice of determining BAC using aorta and not peripheral blood may also account for differences between Garriott’s results and those found in the present study.

To the best of our knowledge this is the first correlation study between abdominal ethanol level and BAC. The high degree of correlation obtained confirms the suitability of muscle as an alternative matrix to assess alcohol impairment when post-mortem blood is not available. Moreover, measuring the ethanol content in muscle samples can be of great utility in cases with unusual post-mortem redistribution [35], and can help discriminate between ante-mortem ethanol intake and post-mortem formation/production [36]. Further studies are required to verify the influence of possible ethanol absorption in traumatisms involving stomach rupture or due to post-mortem ethanol production in bodies found in advanced decomposition state.

## Conclusions

Alcohol-induced alterations were evaluated in three cases of death characterized by severe injury, through toxicological analyses on abdominal muscle, the only available biological matrix. The correlation study between BAC and muscle ethanol level demonstrated the potential of the latter as a post-mortem matrix to assess an impairment when blood is not available.
